# Next-Generation Sequencing of *PTGS* Genes Reveals an Increased Frequency of Non-synonymous Variants Among Patients With NSAID-Induced Liver Injury

**DOI:** 10.3389/fgene.2019.00134

**Published:** 2019-02-28

**Authors:** María Isabel Lucena, Elena García-Martín, Ann K. Daly, Miguel Blanca, Raúl J. Andrade, José A. G. Agúndez

**Affiliations:** ^1^Unidad de Gestión Clínica de Aparato Digestivo, Servicio de Farmacología Clínica, Centro de Investigación Biomédica en Red de Enfermedades Hepáticas y Digestivas Málaga, Instituto de Investigación Biomédica de Málaga, Hospital Universitario Virgen de la Victoria, Universidad de Málaga, Málaga, Spain; ^2^Instituto de Salud Carlos III, University Institute of Molecular Pathology Biomarkers, UNEx, ARADyAL, Cáceres, Spain; ^3^Liver Research Group, Institute of Cellular Medicine, The Medical School, Newcastle University, Newcastle upon Tyne, United Kingdom; ^4^Servicio de Alergología, Hospital Infanta Leonor, ARADyAL, Madrid, Spain

**Keywords:** PTGS1, PTGS2, next generation sequencing, drug-induced liver injury, COX1, COX2

## Abstract

**Purpose:** The etiopathogenesis of drug-induced liver injury (DILI) is still far from being elucidated. This study aims to the study of genetic variations in DILI, related to the drug target, and specifically in the genes coding for the cyclooxygenase enzymes.

**Methods:** By using Next-generation Sequencing we analyzed the genes coding for COX enzymes (*PTGS1* and *PTGS2*) in 113 individuals, 13 of which were patients with DILI caused by COX-inhibitors.

**Results:** The key findings of the study are the increased frequency, among DILI patients, of SNPs causing alterations in transcription factor binding sites and non-synonymous *PTGS* gene variants, as compared to control subjects. Moreover, the association with non-synonymous SNPs was exclusive of DILI patients with late-onset (50 days or more) Pc < 0.001 as compared to DILI patients with early onset, or with control subjects.

**Conclusions:** Our findings suggest an interaction of long-term exposure to COX inhibitors combined with functional variants of the COX enzymes in the risk of developing DILI. This is a novel observation that might have been overlooked by previous genetic studies on DILI because of the limited coverage of PTGS genes in exome chips.

## Background

Although drug-induced liver injury (DILI) is a rare adverse drug event, it is often life-threatening because of the risk of developing acute liver failure. The mechanisms underlying DILI risk are not well understood and hence, the search for biomarkers of DILI risk is a major research field that aims to identify markers that could be used as both proof of the mechanisms involved and of the risk factors that can be used for DILI prediction, as has already been done with many pharmacogenomics biomarkers (Lucena et al., 2008, 2010; Agúndez, 2009; Agundez et al., 2009, 2011; Andrade et al., 2009; Robles-Diaz et al., 2016; Nicoletti et al., 2017). There are presently several independent hypotheses to explain idiosyncratic DILI, but none of these is able to explain all the circumstances in which DILI occurs.

Some genetic biomarkers for DILI either mechanistically-based using a case-control strategy or with a GWAs/exome sequencing approaches have been identified [for a review, see (Robles-Diaz et al., 2016)]. However, the involvement of genetic changes in DILI risk (for instance HLA risk alleles) has been documented for only a few drugs (Kaliyaperumal et al., 2018). On the other hand, case-control genotyping studies, GWAS and exome sequencing have important limitations because only some SNPs are tested, and most of the target sequence is not checked. To overcome this problem, deep sequencing comprising whole genes is necessary.

In this study, we analyzed the potential effect of mutations in cyclooxygenase genes (*PTGS1* and *PTGS2*) on DILI risk related to NSAIDs. From a mechanistic point of view, such a risk could be related to genetic alteration in the arachidonic acid pathways, which are closely related to inflammation. On the other hand, adverse drug events for drugs acting on the COX enzymes (that is, COX inhibitors) may be more likely if COX activity is altered because of genetic variations. For this reason, we analyzed patients who developed DILI after the administration of COX-inhibitors and healthy individuals who tolerated COX-inhibitors.

## Case Presentation

Thirteen patients (8 women and 5 men) who experienced DILI caused by COX inhibitors and 100 individuals who tolerated COX-inhibitors at standard doses were included in this study. The culprit drug for DILI and clinical details of patients are shown in Table 1. Gender-matched control individuals who tolerated COX-inhibitors (62 women and 38 men) individuals were recruited among staff and medical students of the Hospitals and the Universities participating in this study. Individuals which were considered as healthy after medical examination, to exclude pre-existing disorders and history of adverse events after the use of COX-inhibitors, were asked to participate and over 95% of these agreed to do so. We selected consecutive control subjects matched with patients for drug exposure: Fifty control subjects who have received ibuprofen within the previous month to sample collection, 20 who received diclofenac, 10 indomethacin, 10 naproxen, and 10 rofecoxib. These frequencies match with the frequencies for the DILI patients, except that no control subject received nimesulide since this drug was discontinued from the Spanish market due to liver safety. Both patients and controls were Caucasian Spanish individuals. Written informed consent for participation in this case report was obtained from all participants. The protocol for this study was in accordance with the Declaration of Helsinki and its subsequent revisions and was approved by the respective Ethics Committees of the participating Hospitals.

**Table 1:** Demographic and clinical characteristics of 13 patients with NSAIDs-induced idiosyncratic liver injury.

**N^**°**^**	**Culprit drug**	**Age/Sex**	**Indication**	**Daily dose (mg)**	**Duration treatment (days)**	**Time to onset (days)**	**Associated medications**	**Clinical presentation**	**Type of liver injury**	**TB (xULN)**	**ALT (xULN)**	**ALP (xULN)**	**Severity index**	**Outcome**	**CIOMS score**
1	Diclofenac	76/F	Lumbar Pain	50	93	30	Levothyroxine Metamizole	Jaundice ASMA 1/80	HC	2.9	24	2.6	Moderate	Resolution 221 days	Highly probable
2	Nimesulide	62/F	Osteoarthritis	200	30	30	None	Jaundice Eosinophilia	HC	24	98	2.5	Moderate	Resolution 90 days	Probable
3	Nimesulide	61/F	Osteoarthritis	200	62	62	Fosinopril Torasemide	Jaundice	HC	15	30	1.7	Moderate, hospitalized	Resolution 126 days	Highly probable
4	Indometacin	54/F	Osteoarthritis	100	11	8	Tramadol Tetrazepam Dexamethasone	Elevated liver enzymes	Mix	1	16	3.6	Mild	Resolution 44 days	Highly probable
5	Ibuprofen	64/F	Pain	600	17	10	Enalapril Insulin	Jaundice ASMA 1/40	Mix	9	5.9	1.7	Moderate, hospitalized	Resolution 180 days	Highly probable
6	Naproxen	65/M	Pain	1,000	44	48	Tetrazepam Ranitidine Heparin Bisoprolol Metamizole	Jaundice	Mix	4.5	9.7	3.4	Moderate, hospitalized	Lost of follow-up	Highly probable
7	Ibuprofen	18/M	Pain	600	1	2	None	Jaundice Lymphopenia	Mix	7.9	5.5	2.6	Moderate, hospitalized	Lost of follow-up	Highly probable
8	Rofecoxib	82/M	Osteoarthritis	12.5	60	59	Nimodipine Omeprazole Paracetamol Troxerutin Insulin Vitamins Folic Acid	Lymphopenia	Chol	2.6	4.9	4.4	Moderate	Resolution 94 days	Highly probable
9	Ibuprofen	57/M	Pain	1,200	31	50	Glibenclamide	Elevated liver enzymes	HC	1	24	0.9	Mild	Lost of follow-up	Highly probable
10	Nimesulide	59/F	Osteoarthritis	200	25	466	Atenolol Captopril Hydrochlorothiazide Insulin Metformin	Eosinophilia	Chol	1.2	1.2	5	Moderate, hospitalized	Resolution 284 days	Highly probable
11	Ibuprofen	43/M	Pain	1,200	8	8	Metronidazole	Jaundice	HC	1.9	8.2	1.3	Mild, hospitalized	Resolution 308	Probable
12	Diclofenac	80/F	Pain	75	171	171	Telmisartan Allopurinol Glibenclamide Ebastine Troxerutin	Jaundice	HC	4.9	38	1.9	Moderate, hospitalized	Resolution 348	Highly probable
13	Ibuprofen	41/F	Rheumatoid arthritis	600	3	7	Diclofenac Paracetamol Diazepam Fluoxetine	Elevated liver enzymes	HC	1.6	14	1.8	Mild	Resolution 31	Probable

*F, Female; M, Male; ASMA, anti-smooth muscle autoantibody; HC, Hepatocellular, Mix, mixed; Chol, cholestatic; TB, total bilirubin; ALT, alanine aminotransferase; ALP, alkaline phosphatase; ULN, upper limit of normal. Severity index, Mild, elevated ALT/ALP meeting DILI criteria with total bilirubin < 2 mg/dL; Moderate, elevated ALT/ALP with total bilirubin ≥ 2 g/dL; Severe, elevated ALT/ALP and one of the following, ascites, encephalopathy, international normalization ratio >1,5 and/or other organ failure considered to be due to DILI; Fatal, death or transplantation due to DILI*.

## Description Of Laboratory Investigations And Diagnostic Tests

To achieve complete gene capture, we sequenced all exons, intron-exon boundaries as well as the 5′ and 3′ flanking regions for both genes. Referred to the GRCh37 assembly of the human genome, the sequences studied were the following: *PTGS1*: Chromosome 9:125.131.159 to 125.158.017; *PTGS2*: Chromosome 1:186.640.825 to 186.651.605. Partially overlapping amplicons with a size lower than 400 bp were designed. A total of 62 CS1/CS2 tagged primer pairs were synthesized and used to amplify 113 DNA samples using the Access Array platform (Fluidigm). During amplification, samples were labeled with standard MID barcodes designed for the FLX454 sequencing system. After amplification and MID-labeling, individual amplicon libraries were analyzed using a Bioanalyzer 2100 (Agilent) and bioanalyzer traces were used to estimate the amplicon concentration for each sample. Samples were then pooled, and libraries were purified by SPRI using Ampure beads to remove all possible traces of small molecules, primers, primer-dimers, or any other contaminants. The pooled library was again quantified and titrated so that a final amount of 1.95E+10 molecules with an enrichment percentage of 7% was loaded on a Pico Titer Plate (Roche) for a 200-cycle titanium-based sequencing run, made on FLX-454 equipment. Reads were processed using an amplicon processing pipeline and sff files were used for further analyses. Coverage averaged around 50x for the whole project. Coverage for the SNPs identified (shown in Supplemental Table 1) was always over 50x. Sequencing reads were de-multiplexed and aligned using the Amplicon Variant Analyzer software v2.8 (Roche) so that reads for each particular sample- target region combination were analyzed in search of variants. Details of the amplification and sequencing primers are available in Supplemental Table 1.

The putative effect on the non-synonymous variants identified *in silico* was assessed by using the Sorting Tolerant form Intolerant (SIFT) and Polymorphism Phenotyping (PolyPhen) scores as shown in the 1,000 genomes website for every SNP, as well as the online application MutationAssessor (http://mutationassessor.org/r3/).

## Results

The sequencing results (summarized in Table 2) reveal that *PTGS* genes are well conserved. Although dozens of *PTGS1* and *PTGS2* single nucleotide polymorphisms (SNPs) have been described to occur in Caucasian populations (see Agúndez et al., 2015), our findings show that most of these SNPs were not identified, or were extremely rare, in this cohort. Interestingly, most of the *PTGS1* and *PTGS2* SNPs included in the Illumina human exome chip or human core exome chip (Urban et al., 2012) are also absent in this study group. This raises doubts about the coverage of exome chips to identify genetic associations related to *PTGS1* and *PTGS2* genes.

**Table 2:** *PTGS1* and *PTGS2* variant sequences identified in the study group.

**Coordinate GRCh37.p13 (GCA_000001405.14)**	**rs ID**	**N^**°**^ case**	**1**	**2**	**3**	**4**	**5**	**6**	**7**	**8**	**9**	**10**	**11**	**12**	**13**	**Effect**	**MAF DILI**	**MAF control**	**MAF IBS**	**MAF AFR**	**MAF AMR**	**MAF** **EAS**	**MAF EUR**	**MAF SAS**
**PTGS1 (COX-1)**																								
9:125131480	rs10306108		0	0	0	0	0	0	1	0	1	0	0	0	0	UGV	0.077	0.055	0.06	0.13	0.03	0.00	0.07	0.01
9:125131631	rs10306109		0	0	0	0	0	0	1	0	1	0	0	0	0	UGV	0.077	0.055	0.06	0.13	0.03	0.00	0.07	0.01
9:125131688	rs1330344		0	0	0	0	0	1	1	0	1	0	1	0	0	UGV	0.154	0.210	0.24	0.52	0.21	0.42	0.20	0.39
9:125131832	rs10306225		0	0	0	0	0	0	0	2	0	2	0	0	0	UGV	0.154	0.000	0.00	0.00	0.00	0.00	0.01	0.00
9:125132027	rs115693689		0	0	0	0	0	0	1	0	0	0	0	0	0	UGV	0.038	0.055	0.07	0.13	0.04	0.05	0.08	0.02
9:125132028	rs114079139		0	0	0	0	0	0	1	0	0	0	0	0	0	UGV	0.038	0.055	0.07	0.13	0.04	0.05	0.08	0.02
9:125132069	rs77676149		0	0	0	0	0	0	0	1	0	1	0	0	0	UGV	0.077	0.000	0.00	0.01	0.01	0.00	0.00	0.00
9:125132223	rs75993350		0	0	0	0	0	0	0	0	1	0	0	0	0	UGV	0.038	0.055	0.06	0.13	0.03	0.00	0.07	0.01
9:125132311	rs10306110		0	0	0	0	0	0	0	0	1	0	0	0	0	UGV	0.038	0.055	0.06	0.13	0.03	0.00	0.07	0.01
9:125132522	rs10306114		0	0	0	0	0	0	0	0	1	0	0	0	0	UGV	0.038	0.055	0.06	0.13	0.03	0.00	0.07	0.01
9:125132909	rs10306115		0	0	0	0	0	0	2	0	0	0	0	0	0	5′RRV	0.077	0.000	0.00	0.07	0.00	0.00	0.00	0.00
9:125133479	rs1236913		0	0	1	0	0	0	0	1	0	0	0	1	0	MSV^a^	0.115	0.065	0.07	0.01	0.07	0.01	0.06	0.18
9:125133507	rs3842787		0	0	1	0	0	0	0	0	0	0	0	1	0	MSV^b^	0.077	0.060	0.06	0.15	0.03	0.00	0.07	0.01
9:125140206	rs3842788		0	0	0	0	0	0	1	0	0	0	0	0	0	SV	0.038	0.025	0.02	0.32	0.03	0.07	0.04	0.04
9:125140287	rs3842790		0	0	0	0	0	0	1	0	0	0	0	0	0	SV	0.038	0.000	0.00	0.06	0.00	0.00	0.00	0.00
9:125140696	rs2282169		0	1	0	1	0	0	2	0	0	0	1	0	0	IV	0.192	0.140	0.15	0.60	0.28	0.11	0.19	0.23
9:125140823	rs5787		0	0	0	0	0	0	0	0	0	2	0	0	0	MSV^c^	0.077	0.000	0.00	0.00	0.00	0.00	0.00	0.00
9:125141239	rs12555242		0	0	0	0	0	0	0	0	0	0	2	0	0	IV	0.077	0.070	0.02	0.00	0.03	0.05	0.02	0.08
9:125143707	rs3842792		0	0	0	0	0	0	0	0	0	0	0	1	0	MSV^d^	0.038	0.000	0.00	0.04	0.00	0.00	0.00	0.00
9:125143792	rs5788		0	1	0	1	0	0	1	0	0	0	0	0	0	SV	0.115	0.110	0.11	0.69	0.24	0.04	0.14	0.10
9:125143882	rs3842794		0	0	0	0	0	0	0	1	0	1	0	0	0	IV	0.077	0.000	0.00	0.02	0.00	0.00	0.00	0.00
9:125144040	rs3215925		0	1	0	1	0	0	1	0	0	0	0	0	0	IV	0.115	0.095	0.11	0.67	0.24	0.04	0.14	0.10
9:125145743	rs3842798		0	1	0	1	0	2	0	0	0	0	2	0	0	IV	0.231	0.170	0.15	0.76	0.30	0.11	0.20	0.23
9:125155408	rs8046		0	0	0	0	0	0	0	0	0	0	1	0	0	3′UTRV	0.038	0.000	0.04	0.51	0.10	0.09	0.07	0.14
9:125155930	rs10306192		0	0	0	0	0	0	0	0	0	0	2	0	0	3′UTRV	0.077	0.000	0.04	0.01	0.05	0.09	0.06	0.13
9:125156374	rs199981440		0	0	0	0	0	0	0	1	0	0	0	1	0	3′UTRV	0.077	0.000	–	–	–	–	–	–
9:125157198	rs10306194		0	0	1	0	1	0	2	0	0	1	2	0	0	3′UTRV	0.269	0.175	0.20	0.02	0.11	0.04	0.15	0.14
9:125157316	rs10306196		0	0	0	0	0	0	0	0	0	0	1	0	0	3′UTRV	0.038	0.065	0.04	0.01	0.05	0.09	0.06	0.13
9:125157357	rs10306197		0	0	0	0	0	0	1	0	0	0	0	0	1	3′UTRV	0.077	0.000	0.00	0.02	0.00	0.00	0.00	0.00
9:125157672	rs10306199		0	0	0	0	1	1	0	0	0	0	0	0	0	3′UTRV	0.077	0.000	0.00	0.02	0.00	0.00	0.00	0.00
9:125157718	rs9233		0	0	0	0	0	0	0	0	0	0	1	0	0	3′UTRV	0.038	0.065	0.04	0.01	0.05	0.09	0.06	0.14
**PTGS2 (COX-2)**																								
1:186640853	rs4648304		1	0	0	0	0	0	0	0	0	0	0	0	0	3′UTRV	0.038	0.000	0.00	0.10	0.01	0.00	0.00	0.00
1:186641058	rs689470		1	0	1	0	0	0	0	0	0	0	0	0	0	3′UTRV	0.077	0.000	0.05	0.50	0.05	0.01	0.03	0.07
1:186641265	rs689468		0	0	1	0	0	0	0	0	0	0	0	0	0	3′UTRV	0.038	0.005	0.03	0.00	0.01	0.01	0.02	0.03
1:186641273	rs689467		0	0	0	0	0	0	0	0	0	1	0	0	0	3′UTRV	0.038	0.025	0.07	0.00	0.03	0.00	0.05	0.03
1:186641577	rs4648299		0	0	0	0	0	0	0	1	0	0	0	0	0	3′UTRV	0.038	0.000	0.00	0.00	0.00	0.00	0.00	0.00
1:186641682	rs4648298		0	0	1	0	0	0	0	0	0	0	0	0	0	3′UTRV	0.038	0.005	0.03	0.00	0.01	0.00	0.02	0.03
1:186642059	rs4648297		0	0	0	0	0	0	0	1	0	0	0	0	0	3′UTRV	0.038	0.000	0.00	0.00	0.00	0.00	0.00	0.00
1:186642429	rs2206593		0	0	0	0	0	0	0	0	1	0	0	0	0	3′UTRV	0.038	0.050	0.06	0.00	0.05	0.00	0.09	0.06
1:186642856	rs4648292		0	0	0	0	0	0	0	1	1	0	0	0	0	3′UTRV	0.077	0.000	0.00	0.08	0.00	0.00	0.00	0.00
1:186642987	rs5276		1	0	0	0	0	0	0	0	0	0	0	0	0	3′UTRV	0.038	0.015	0.00	0.18	0.01	0.00	0.00	0.00
1:186643058	rs5275		1	0	1	0	0	1	1	1	0	0	0	1	1	3′UTRV	0.269	0.205	0.33	0.64	0.37	0.20	0.31	0.40
1:186643204	rs36233646		1	0	0	0	0	0	0	1	0	0	0	0	0	3′UTRV	0.077	0.000	0.02	0.47	0.04	0.00	0.01	0.04
1:186643238	rs4648290		0	0	0	0	0	0	0	0	1	0	0	0	0	3′UTRV	0.038	0.010	0.00	0.00	0.01	0.00	0.01	0.00
1:186643803	rs4648288		0	0	0	0	0	0	0	0	0	0	0	1	0	SV	0.038	0.000	0.00	0.00	0.00	0.00	0.00	0.00
1:186645078	rs5279		0	0	0	0	0	0	0	0	1	0	0	0	0	SV	0.038	0.005	0.00	0.08	0.00	0.00	0.00	0.00
1:186645669	rs5278		0	0	0	0	0	0	0	0	1	0	0	0	0	SV	0.038	0.000	0.00	0.08	0.00	0.00	0.00	0.00
1:186645927	rs2066826		0	0	0	0	0	0	0	0	1	0	0	0	0	IV	0.038	0.105	0.12	0.36	0.19	0.04	0.12	0.16
1:186646005	rs3218622		0	0	0	0	0	0	0	0	1	0	0	0	0	MSV^e^	0.038	0.000	0.00	0.00	0.00	0.00	0.00	0.00
1:186647418	rs4648265		0	0	0	0	0	0	0	0	0	0	1	0	0	SV	0.038	0.000	0.00	0.00	0.00	0.00	0.00	0.00
1:186648157	rs2066823		0	0	0	0	0	0	0	0	0	1	0	0	0	IV	0.038	0.000	0.00	0.00	0.00	0.00	0.00	0.00
1:186648197	rs5277		0	1	1	0	0	0	0	2	0	0	2	2	1	IV	0.346	0.180	0.15	0.01	0.11	0.04	0.18	0.06
1:186650163	rs20419		1	0	0	0	0	0	0	0	0	0	0	0	0	RRV	0.038	0.000	0.00	0.10	0.01	0.00	0.00	0.00
1:186650214	rs148416467		0	0	0	0	0	0	0	2	0	0	0	0	0	RRV	0.077	0.000	0.15	0.08	0.00	0.00	0.00	0.00
1:186650321	rs20417		0	0	1	0	0	0	0	0	0	2	0	0	1	RRV	0.154	0.190	0.00	0.35	0.21	0.04	0.15	0.19
1:186650688	rs20415		1	0	0	0	0	0	0	0	0	0	0	0	0	RRV	0.038	0.000	0.15	0.10	0.01	0.00	0.00	0.00
1:186650751	rs689466		1	0	0	1	0	0	0	0	0	0	0	0	0	RRV	0.077	0.215	0.15	0.08	0.26	0.48	0.19	0.13
1:186650846	rs689465		0	0	0	0	0	0	0	0	0	2	0	0	2	RRV	0.154	0.235	0.11	0.16	0.18	0.05	0.13	0.16
1:186650857	rs4648253		0	1	0	0	0	0	0	0	0	0	0	0	0	RRV	0.038	0.000	0.00	0.00	0.00	0.00	0.00	0.01
1:186650877	rs72366725		0	0	1	0	0	0	0	0	0	0	0	0	0	RRV	0.038	0.005	0.05	0.27	0.04	0.00	0.03	0.03
1:186651296	rs4648250		0	0	0	0	0	0	0	0	0	2	0	0	0	RRV	0.077	0.015	0.00	0.00	0.07	0.03	0.01	0.07
1:186651571	unknown		0	1	0	0	0	0	0	0	0	0	0	0	0	RRV	0.038	0.000	–	–	–	–	–	–

MAF, Minor allele frequency referred to the GRCh37 assembly as shown in the 1,000 genomes website http://phase3browser.1000genomes.org/Homo_sapiens/Info/Index. 0, Non-mutated; 1, heterozygous; 2, Homozygous for the minor allele; UGV, Upstream gene variant; RRV, Regulatory region variant.; MSV, Missense variant; SV, Synonymous variant; IV, INtron variant; UTRV, Untranslated region variant; Populations, IBS correspond to the Iberian Populations in Spain (a subpopulation of Europeans); AFR, Africans; AMR, Ad mixed americans; EAS, East asians; EUR, Europeans; SAS, South asians, as described in detail in the website http://phase3browser.1000genomes.org/Help/Faq?id=328. Predicted consequences for missense variants:

a *Nonsynonymous (W8R), SIFT score = 0.85 (tolerated, low confidence), PolyPhen score = 0 (unknown), Mutation Assessor = neutral*.

b *Nonsynonymous (P17L), SIFT score = 1.00 (tolerated, low confidence), PolyPhen score = 0 (unknown), Mutation Assessor = low impact*.

c *Nonsynonymous (R108Q), SIFT score = 0.12 (tolerated), PolyPhen score = 0.21 (benign), Mutation Assessor = medium impact*.

d *Nonsynonymous (K185T), SIFT score = 0.36 (tolerated), PolyPhen score = 0.007 (benign), Mutation Assessor = neutral*.

e *Nonsynonymous (R228H), SIFT score = 1.00 (tolerated), PolyPhen score = 0.002 (benign), Mutation Assessor = neutral*.

In the whole population study, we identified 31 single nucleotide polymorphisms (SNPs) for *PTGS1*, including four non-synonymous SNPs. For *PTGS2* we identified 31 SNPs including one non-synonymous. We observed an increased frequency of *PTGS1* and *PTGS2* mutations among DILI patients, as compared to that observed in control individuals. Most of the SNPs identified in patients were rare among control individuals and were rare also according to the 1,000 genomes database (as shown in Table 2). All patients but one (case 1 in Table 2) had mutations at the *PTGS1* gene and all patients but one (case 5 in Table 2) had mutations at the *PTGS2* gene. Table 3 summarizes the comparison of relevant SNPs across patients with late-onset DILI, the rest of DILI patients and control individuals.

**Table 3:** Detailed genotype distribution for relevant SNPs.

**Coordinate GRCh37.p13 (GCA_000001405.14)**	**rs ID**	**Effect**	**Patients with late-onset DILI** ***N* = 5** **(Non carriers / heterozygous/ homozygous); MAF**	**Rest of DILI patients** ***N* = 8** **(Non carriers / heterozygous/ homozygous); MAF**	**Control individuals** ***N* = 100** **(Non carriers / heterozygous/ homozygous); MAF**
9:125131832	rs10306225	UGV	3/0/2; 0.400	8/0/0; 0.000	100/0/0; 0.000
9:125133479	rs1236913	MSV	2/3/0; 0.300	8/0/0; 0.000	88/11/1; 0.065
9:125133507	rs3842787	MSV	3/2/0; 0.200	8/0/0; 0.000	89/10/1; 0.060
9:125140823	rs5787	MSV	4/0/1; 0.200	8/0/0; 0.000	100/0/0; 0.000
9:125143707	rs3842792	MSV	4/1/0; 0.100	8/0/0; 0.000	100/0/0; 0.000
1:186646005	rs3218622	MSV	4/1/0; 0.100	8/0/0; 0.000	100/0/0; 0.000

*MAF, Minor allele frequency; UGV, Upstream gene variant; MSV, Missense variant*.

## Discussion of the Underlying Pathophysiology and the Novelty or Significance of the Case

The most remarkable findings in this study are the presence among DILI patients of SNPs causing alterations in transcription factor binding sites such as the *PTGS1* SNP rs10306225 (Agundez et al., 2014), and the *PTGS2* SNPs rs4648253, rs689466, and rs20417, as well as non-synonymous SNPs such as *PTGS1* rs1236913 (W 8 R), rs3842787 (P 17 L), rs5787 (R 108 Q), rs3842792 (K 185 T), and *PTGS2* rs3218622 (R 228 H). These missense variants are extremely rare among European individuals (Agúndez et al., 2015). The putative effects of the most relevant SNPs shown in Table 3 have been revised elsewhere (Agúndez et al., 2015). In brief, besides the rs10306225 SNP, which is a promoter variant that causes a modification in a CDX1 binding site (Agundez et al., 2014), the rest of SNPs are non-synonymous. According to functional predictions and functional analyses (reviewed in Agúndez et al., 2015) the SNPs rs1236913, rs3842787 have a little functional effect, although clinical associations for these SNPs with urticaria induced by NSAIDs (Cornejo-Garcia et al., 2012) and myocardial infarction/stroke (Lee et al., 2008; Lemaitre et al., 2009; Gao et al., 2014), respectively, have been proposed. The functional effect of the rs5787 SNP is unknown, although functional prediction suggests a mild functional impact (see Table 2), rs3842792 SNP is predicted as functional (Table 2), but *in vitro* findings suggest reduced functionality (Lee et al., 2007), and no functional impact for the *PTGS2* SNP rs3218622 has been described.

No particular association of missense SNPs with culprit drug, age, gender, clinical presentation, type of liver injury, and severity of the disease was identified. However, as shown in Table 1, there is heterogeneity in the duration of treatment before DILI onset. This heterogeneity, rather than being a weakness, is a strong point in this study because it allowed discriminating the frequencies of PTGS gene variations in DILI patients with late and short-term onset. All the five DILI patients with the longest times to DILI onset (50 or more days; patients n° 3, 8, 9, 10, 12 in Table 1) had missense variants, and no patient with shorter time to DILI onset had such missense variants. The intergroup comparison values for carriers of any non-synonymous *PTGS* variants were as follows: Patients with late DILI onset (50 or more days) vs. the rest of DILI patients (*P* < 0.001). Patients with late DILI onset vs. control individuals (*P* < 0.001). By turn, no significant differences for carriers of non-synonymous *PTGS* variants were observed among patients with DILI onset shorter than 50 days and control subjects (*P* = 0.325). Haplotype analyses (Table 4), and linkage disequilibrium (LD) analyses (Supplemental Table 2), show that the risk is due to the presence of rare haplotypes (containing missense variants) in the group of patients with late-onset DILI, but it is not due to LD variations for these variants. The strong association observed in this report, although it is based in five cases only, suggests a relationship of non-synonymous *PTGS* gene variations with DILI onset after long-term NSAID therapy. This is a novel observation that has not been raised by previous studies. Although the putative role of *PTGS* gene variations has been explored using the Illumina human exome chip or human core exome chip, it is of note that chip coverage was very limited for *PTGS* genes (Urban et al., 2012). By turn, this study has complete coverage thus allowing the identification of, as yet, disregarded SNPs. Another relevant difference with most DILI genetic studies is that in this report we stratified patients according to the time to onset. It cannot be ruled out heterogeneity in the etiopathogenesis of DILI, and it is conceivable that the mechanisms involved in DILI with a late onset might be different from those involved in immediate or short-latency reactions. This study, albeit with the inherent limitations of statistical power that case reports have, reinforces the view that a complete gene coverage and a detailed phenotype stratification of DILI patients could be essential to gain strength in further genetic association studies.

**Table 4:** Haplotype analysis.

**Haplotype frequencies**	**rs10306225**	**rs1236913**	**rs3842787**	**rs5787**	**rs3842792**	**rs3218622**	**Frequency (Total)**	**Frequency (late-onset DILI cases)**	**Frequency (controls)**
1	A	T	C	G	A	C	0.9048	0.300	0.935
2	A	C	T	G	A	C	0.0619	0.100	0.060
3	T	T	C	A	A	C	0.0095	0.200	NA
4	A	C	C	G	A	C	0.0048	NA	0.005
5	A	T	C	G	A	T	0.0048	0.100	NA
6	T	C	C	G	A	C	0.0048	0.100	NA
7	T	T	C	G	A	C	0.0048	0.100	NA
8	T	T	C	G	A	C	0.0048	0.100	NA
9	T	T	C	G	A	C	0.0048	0.100	NA
**Haplotype association with late-onset DILI**	**rs10306225**	**rs1236913**	**rs3842787**	**rs5787**	**rs3842792**	**rs3218622**	**Frequency (Total)**	**OR (95% CI)**	***P*****-value**
1	A	T	C	G	A	C	0.9048	1.00	—
2	A	C	T	G	A	C	0.0619	0.09 (0.01–1.43)	0.0910
Rare haplotypes	*	*	*	*	*	*	0.0333	0.00 (0.00–0.09)	0.0024

*Global haplotype association p < 0.0001*.

NA, not applicable; *any nucleotide.

## Author Contributions

ML and EG-M participated in the design of the study, in data acquisition, and in critical revision for important intellectual content. AD, MB, and RA participated in the analysis and interpretation of the data and critical revision for important intellectual content. JA participated in the conception, design, data analysis and interpretation, the drafting of the manuscript and critical revision for important intellectual content. All authors approved the final version of the manuscript and all agree to be accountable for all aspects of the work in ensuring that questions related to the accuracy or integrity of any part of the article are appropriately investigated and resolved.

### Conflict of Interest Statement

The authors declare that the research was conducted in the absence of any commercial or financial relationships that could be construed as a potential conflict of interest.

## Abbreviations

COX: Cyclooxygenase, prostaglandin-endoperoxide synthase
NSAID: Non-steroidal anti-inflammatory drug
DILI: Drug-induced liver injury
GWAS: Genome-wide association study
SNP: Single nucleotide polymorphism
HLA: Human leukocyte antigen
PTGS1: Prostaglandin-Endoperoxide Synthase 1
PTGS2: Prostaglandin-Endoperoxide Synthase 2.
