# Supplementary material for: Next-Generation Sequencing of PTGS Genes Reveals an Increased Frequency of Non-synonymous Variants Among Patients With NSAID-Induced Liver Injury
Source: Front Genet. 2019 Feb 28;10:134. doi: 10.3389/fgene.2019.00134 (PMC6403122; doi:10.3389/fgene.2019.00134)
Supplement: Supplementary file 1 [file Data_Sheet_1.docx]

Supplemental Table 1: Details of the sequencing procedure.

| **Gene** | **Amplicon number** | **Amplicon length (bp)** | **Coordinate: Origin** | **Coordinate: End** | **Forward primer** | **Reverse primer** |
| --- | --- | --- | --- | --- | --- | --- |
| *PTGS1* | PTGS1_1 | 380 | 9:125131159 | 9:125131538 | ACACTGACGACATGGTTCTACAAGGTTTGATCCTCCTCATTCCTTG | TACGGTAGCAGAGACTTGGTCTCCAGTTCCAGCATGGAGCAAT |
| *PTGS1* | PTGS1_2 | 381 | 9:125131449 | 9:125131829 | ACACTGACGACATGGTTCTACACTCTCCTGTATTGCAGCTGTGTT | TACGGTAGCAGAGACTTGGTCTAATGAGGTTCCATCTTCCACTCC |
| *PTGS1* | PTGS1_3 | 372 | 9:125131755 | 9:125132126 | ACACTGACGACATGGTTCTACAGCACAGGTGTGGTGACTTGATG | TACGGTAGCAGAGACTTGGTCTATTCTCACCCAAGCTGTTCCC |
| *PTGS1* | PTGS1_4 | 375 | 9:125132048 | 9:125132422 | ACACTGACGACATGGTTCTACAGTCTGCCCTGGCTTGCTC | TACGGTAGCAGAGACTTGGTCTACATCCACCGCCGAATTTATAG |
| *PTGS1* | PTGS1_5 | 380 | 9:125132333 | 9:125132712 | ACACTGACGACATGGTTCTACATGGACCTATTCCTTCCTCCTTCT | TACGGTAGCAGAGACTTGGTCTTCCAAACTCCAAGAGAGCCTAGT |
| *PTGS1* | PTGS1_6 | 385 | 9:125132609 | 9:125132993 | ACACTGACGACATGGTTCTACAAGATGAGAATACTGAGGGCTAGAA | TACGGTAGCAGAGACTTGGTCTCTGCCCCGCAGAGAAGAAAG |
| *PTGS1* | PTGS1_7 | 366 | 9:125132920 | 9:125133285 | ACACTGACGACATGGTTCTACAACCCTTTTCTGTAGAGTGCGTTA | TACGGTAGCAGAGACTTGGTCTATCCCCTCCCCACCCTTC |
| *PTGS1* | PTGS1_8 | 384 | 9:125133199 | 9:125133582 | ACACTGACGACATGGTTCTACAGGCTGGGCAGAGGAAGTAAG | TACGGTAGCAGAGACTTGGTCTGATTCCCTTGGGGAGGGATG |
| *PTGS1* | PTGS1_9 | 384 | 9:125139990 | 9:125140373 | ACACTGACGACATGGTTCTACATGGTTTATGTTCACAGAGGTGGG | TACGGTAGCAGAGACTTGGTCTAAGTTAGGGTCTAGGAGAAAGGG |
| *PTGS1* | PTGS1_10 | 329 | 9:125140658 | 9:125140986 | ACACTGACGACATGGTTCTACATACTTCTGGTTCTGGTAGGAGGG | TACGGTAGCAGAGACTTGGTCTCTCCTGGGTTGGGGCTTATTTTA |
| *PTGS1* | PTGS1_11 | 353 | 9:125140920 | 9:125141272 | ACACTGACGACATGGTTCTACAGCATCTGATAAGGGTAGTGGGTG | TACGGTAGCAGAGACTTGGTCTTCGCCTGATTTTTCCCAAAGAGA |
| *PTGS1* | PTGS1_12 | 382 | 9:125143496 | 9:125143877 | ACACTGACGACATGGTTCTACAAGAGAGTGGGTTACTTGGTGGT | TACGGTAGCAGAGACTTGGTCTGCCAAATTAGGTCCAGAGCAGT |
| *PTGS1* | PTGS1_13 | 357 | 9:125143733 | 9:125144089 | ACACTGACGACATGGTTCTACAAACCTCATGTTTGCCTTCTTTGC | TACGGTAGCAGAGACTTGGTCTGTCTTTGCCAGGGAAGACCAT |
| *PTGS1* | PTGS1_14 | 362 | 9:125145716 | 9:125146077 | ACACTGACGACATGGTTCTACATGGGAGGGAGTTGGTTGT | TACGGTAGCAGAGACTTGGTCTGAGGGAATGACCTTCCAGGG |
| *PTGS1* | PTGS1_15 | 382 | 9:125148658 | 9:125149039 | ACACTGACGACATGGTTCTACAGCTTTCCAAGCTGGGCATCTA | TACGGTAGCAGAGACTTGGTCTCCCTCACCAGCACTCCTCTG |
| *PTGS1* | PTGS1_16 | 341 | 9:125152354 | 9:125152694 | ACACTGACGACATGGTTCTACACCAACACTCTCCATCCTAGCTCA | TACGGTAGCAGAGACTTGGTCTCTTGATGTCTCCCTGACTTTGCT |
| *PTGS1* | PTGS1_17 | 371 | 9:125154403 | 9:125154773 | ACACTGACGACATGGTTCTACACGCCCCAGGTTGACCTTAAT | TACGGTAGCAGAGACTTGGTCTCATCCGGCACACGGAAGG |
| *PTGS1* | PTGS1_18 | 362 | 9:125154704 | 9:125155065 | ACACTGACGACATGGTTCTACAGCCACACTGAAGAAGCTGGTC | TACGGTAGCAGAGACTTGGTCTGGCAGACCAAATGAGAACCACTC |
| *PTGS1* | PTGS1_19 | 353 | 9:125155012 | 9:125155364 | ACACTGACGACATGGTTCTACATGAACTCCTTGTTAGCCCTTCAG | TACGGTAGCAGAGACTTGGTCTTTGTTGGACCTTAGGATCTCCGT |
| *PTGS1* | PTGS1_20 | 371 | 9:125155273 | 9:125155643 | ACACTGACGACATGGTTCTACAATCTGTCAGCATCTGGCTGTCTA | TACGGTAGCAGAGACTTGGTCTCTAGCCATGTTCCAGTGTTGCC |
| *PTGS1* | PTGS1_21 | 359 | 9:125155457 | 9:125155815 | ACACTGACGACATGGTTCTACATAGAGAGAACAGGTGGGCTGTAT | TACGGTAGCAGAGACTTGGTCTGGTCTGGGGCAATGGGTATTTTA |
| *PTGS1* | PTGS1_22 | 379 | 9:125155762 | 9:125156140 | ACACTGACGACATGGTTCTACAGCCAGGCACTGCCCTTT | TACGGTAGCAGAGACTTGGTCTACTCCACCATTCTGTCTTTGACC |
| *PTGS1* | PTGS1_23 | 315 | 9:125156058 | 9:125156372 | ACACTGACGACATGGTTCTACATGCTCCTCTTGATTCCTGGTTTG | TACGGTAGCAGAGACTTGGTCTGGGTTAGAAGGGAAGTCACACTC |
| *PTGS1* | PTGS1_24 | 382 | 9:125156138 | 9:125156519 | ACACTGACGACATGGTTCTACAAGTGTAGCCTCCACCTGATATTC | TACGGTAGCAGAGACTTGGTCTCATCTTACCCCTCTGTCCTCTCT |
| *PTGS1* | PTGS1_25 | 369 | 9:125156463 | 9:125156831 | ACACTGACGACATGGTTCTACAGACCCTTTTCTCAGGACCTCTGT | TACGGTAGCAGAGACTTGGTCTATCTGCAAGAAAGGACAGGACAC |
| *PTGS1* | PTGS1_26 | 384 | 9:125156751 | 9:125157134 | ACACTGACGACATGGTTCTACATGCAGTTGCTCTGACGTAGAAAG | TACGGTAGCAGAGACTTGGTCTACCGGATGGAAGGAAAAGCTAAA |
| *PTGS1* | PTGS1_27 | 378 | 9:125157046 | 9:125157423 | ACACTGACGACATGGTTCTACAAGATGGTCCATTTGTTCCTGCTT | TACGGTAGCAGAGACTTGGTCTCTCAAAGCCCTTCCCCTAGC |
| *PTGS1* | PTGS1_28 | 383 | 9:125157335 | 9:125157717 | ACACTGACGACATGGTTCTACAAAGGACAGGAAGCTGGCAGAA | TACGGTAGCAGAGACTTGGTCTATACCTTCACAGCCACACCTAGA |
| *PTGS1* | PTGS1_29 | 385 | 9:125157633 | 9:125158017 | ACACTGACGACATGGTTCTACATGGTTGGTTTTATTTGTCAGTTTGGT | TACGGTAGCAGAGACTTGGTCTGATCCTGAAACAAATCCCCCACA |
| *PTGS2* | PTGS2_1 | 369 | 1:186640825 | 1:186641193 | ACACTGACGACATGGTTCTACACATGAAGGGCCAGTCCTAGTTTT | TACGGTAGCAGAGACTTGGTCTAGAATGTTTAAGGTTAAGAAAGAAATAGTCA |
| *PTGS2* | PTGS2_2 | 383 | 1:186641069 | 1:186641451 | ACACTGACGACATGGTTCTACACCAAATTCCCAGGTTTTGTCAGC | TACGGTAGCAGAGACTTGGTCTTGAATGTGCCATAAGACTGACCT |
| *PTGS2* | PTGS2_3 | 293 | 1:186641236 | 1:186641528 | ACACTGACGACATGGTTCTACATGATATTTATTTCATTTTCTCCCTCTTCC | TACGGTAGCAGAGACTTGGTCTTGAGATATTTAAGGTTGAATGTTTGTCCT |
| *PTGS2* | PTGS2_4 | 366 | 1:186641429 | 1:186641794 | ACACTGACGACATGGTTCTACAAGGTCAGTCTTATGGCACATTCA | TACGGTAGCAGAGACTTGGTCTACGATAATACTTCTTTTCCACATCTCA |
| *PTGS2* | PTGS2_5 | 382 | 1:186641514 | 1:186641895 | ACACTGACGACATGGTTCTACAACCTTAAATATCTCAATATGCCAATCAG | TACGGTAGCAGAGACTTGGTCTTGTGACTGGAAAAAGTTACGTTCC |
| *PTGS2* | PTGS2_6 | 342 | 1:186641757 | 1:186642098 | ACACTGACGACATGGTTCTACAGTCAGTGACAATGAGATGTGGAA | TACGGTAGCAGAGACTTGGTCTCAAGTTTTCAGGTAAACCTCAGC |
| *PTGS2* | PTGS2_7 | 335 | 1:186641920 | 1:186642254 | ACACTGACGACATGGTTCTACACACTAGCCTCTTTGCATCCATCT | TACGGTAGCAGAGACTTGGTCTTACCTGCATGCTGTTCCTTTTCT |
| *PTGS2* | PTGS2_8 | 390 | 1:186642072 | 1:186642461 | ACACTGACGACATGGTTCTACACTGAGCTGAGGTTTACCTGAAA | TACGGTAGCAGAGACTTGGTCTGGTTAATGAAGTACCAAGCTGTGC |
| *PTGS2* | PTGS2_9 | 383 | 1:186642367 | 1:186642749 | ACACTGACGACATGGTTCTACATTTGCAATGTGATATGGACTGCT | TACGGTAGCAGAGACTTGGTCTTTTAGTGTGACTGTTAAAACTTCCTTT |
| *PTGS2* | PTGS2_10 | 316 | 1:186642609 | 1:186642924 | ACACTGACGACATGGTTCTACATTTACATGTTACCAGCCATATAAACAAA | TACGGTAGCAGAGACTTGGTCTAGGGTAGAATCACCTGTAAAAGC |
| *PTGS2* | PTGS2_11 | 368 | 1:186642759 | 1:186643126 | ACACTGACGACATGGTTCTACAACTCTTGGTGAAAAAGGAACATCAC | TACGGTAGCAGAGACTTGGTCTTGTTTCCAATGCATCTTCCATGA |
| *PTGS2* | PTGS2_12 | 303 | 1:186642901 | 1:186643203 | ACACTGACGACATGGTTCTACAAGCTTTTACAGGTGATTCTACCCTATG | TACGGTAGCAGAGACTTGGTCTAGAACGAAAGTAAAGATGTTTGAATACT |
| *PTGS2* | PTGS2_13 | 254 | 1:186643107 | 1:186643360 | ACACTGACGACATGGTTCTACATGGAAGATGCATTGGAAACATCG | TACGGTAGCAGAGACTTGGTCTTGCGGAGAAAGGAGTCATACTTG |
| *PTGS2* | PTGS2_14 | 340 | 1:186643284 | 1:186643623 | ACACTGACGACATGGTTCTACATCCAAACTTAACAGCAACAGCAA | TACGGTAGCAGAGACTTGGTCTGGGCTGTCCCTTTACTTCATTCA |
| *PTGS2* | PTGS2_15 | 349 | 1:186643341 | 1:186643689 | ACACTGACGACATGGTTCTACAGTATGACTCCTTTCTCCGCAACA | TACGGTAGCAGAGACTTGGTCTTGGAGAAGTGGGTTTTCAAATCATC |
| *PTGS2* | PTGS2_16 | 344 | 1:186643583 | 1:186643926 | ACACTGACGACATGGTTCTACAGCTCTGGATCTGGAACACTGAAT | TACGGTAGCAGAGACTTGGTCTTGGTTGTATCTCTGTCTTCATCGC |
| *PTGS2* | PTGS2_17 | 384 | 1:186644338 | 1:186644721 | ACACTGACGACATGGTTCTACAACAAACAAACAAAAAACGAAGAAGTTT | TACGGTAGCAGAGACTTGGTCTTTAGTTCTAGGCTGGTGTCCCAT |
| *PTGS2* | PTGS2_18 | 319 | 1:186644963 | 1:186645281 | ACACTGACGACATGGTTCTACATTTCCGTGGCAGAAATTCTAAAG | TACGGTAGCAGAGACTTGGTCTCAACACTTGAGTGGCTATCACTTC |
| *PTGS2* | PTGS2_19 | 347 | 1:186645035 | 1:186645381 | ACACTGACGACATGGTTCTACACAGCAATTTGCCTGGTGAATGA | TACGGTAGCAGAGACTTGGTCTCCAGTTGCTTGAAAGCTTGTGAT |
| *PTGS2* | PTGS2_20 | 372 | 1:186645535 | 1:186645906 | ACACTGACGACATGGTTCTACAACAAAGATAGCACACTAATTTTCCCT | TACGGTAGCAGAGACTTGGTCTTCCCTAAAAATGTGTTCCTTAACTTTT |
| *PTGS2* | PTGS2_21 | 327 | 1:186645770 | 1:186646096 | ACACTGACGACATGGTTCTACACAGGGACTTGAGGAGGGTAGAT | TACGGTAGCAGAGACTTGGTCTAGAAAAACTTCAACAGCAACAAATTAAAA |
| *PTGS2* | PTGS2_22 | 318 | 1:186646708 | 1:186647025 | ACACTGACGACATGGTTCTACATCTCCTTTAATGTTAGCCCTTGACT | TACGGTAGCAGAGACTTGGTCTCAGTCACCATCTCCTTTCTTGAAT |
| *PTGS2* | PTGS2_23 | 265 | 1:186647338 | 1:186647602 | ACACTGACGACATGGTTCTACAAGCAATGCAGCCCGTCTTATAG | TACGGTAGCAGAGACTTGGTCTAAAATAATGGTTGATTTACTTAACAAATGAGAA |
| *PTGS2* | PTGS2_24 | 369 | 1:186648095 | 1:186648463 | ACACTGACGACATGGTTCTACAAGCTTGGAAATATGTTTTTAGATTAGGC | TACGGTAGCAGAGACTTGGTCTTGCTCAACACGTAAGTTTGTCCT |
| *PTGS2* | PTGS2_25 | 328 | 1:186648282 | 1:186648609 | ACACTGACGACATGGTTCTACAACTGTGTTTGGAGTGGGTTTCAG | TACGGTAGCAGAGACTTGGTCTACACTAGAGAATGATTTATTTGCTGTCC |
| *PTGS2* | PTGS2_26 | 385 | 1:186649312 | 1:186649696 | ACACTGACGACATGGTTCTACAGGAAACTCTGCCCGGGTG | TACGGTAGCAGAGACTTGGTCTCACCGGGCTTACGCAATTTTTT |
| *PTGS2* | PTGS2_27 | 379 | 1:186649589 | 1:186649967 | ACACTGACGACATGGTTCTACAGACTGAAAACCAAGCCCATGTGA | TACGGTAGCAGAGACTTGGTCTGATTCCTGGAGAGGAAGCCAAG |
| *PTGS2* | PTGS2_28 | 377 | 1:186649854 | 1:186650230 | ACACTGACGACATGGTTCTACATTTTCTTCTTCGCAGTCTTTGCC | TACGGTAGCAGAGACTTGGTCTGCTATGTACACTGAAGGTAGCTATT |
| *PTGS2* | PTGS2_29 | 385 | 1:186650126 | 1:186650510 | ACACTGACGACATGGTTCTACACGCTTAATAGGCTGTATATCTGCTC | TACGGTAGCAGAGACTTGGTCTGCATCAGGGAGAGAAATGCCTTA |
| *PTGS2* | PTGS2_30 | 395 | 1:186650411 | 1:186650805 | ACACTGACGACATGGTTCTACATTCCTGCCTTCTGATGGACAAAG | TACGGTAGCAGAGACTTGGTCTCTCCCTGAGCACTACCCATGATA |
| *PTGS2* | PTGS2_31 | 381 | 1:186650664 | 1:186651044 | ACACTGACGACATGGTTCTACACCTGGGCTTATTGGGGCTAA | TACGGTAGCAGAGACTTGGTCTAAAACCTCCAAGTGAGTCTCTTATT |
| *PTGS2* | PTGS2_32 | 400 | 1:186650943 | 1:186651342 | ACACTGACGACATGGTTCTACAGGAAAATGACAGCATAAAACCTGA | TACGGTAGCAGAGACTTGGTCTTGGCTTCTAACCCAAACTAACAT |
| *PTGS2* | PTGS2_33 | 400 | 1:186651206 | 1:186651605 | ACACTGACGACATGGTTCTACAACTAAGAGCGTGGATCATTTTCT | TACGGTAGCAGAGACTTGGTCTTGGAACTCTTCACTCTATCCTGCT |

The coordinates for sequencing correspond to the GRCh37 assembly of the human genome.

Supplemental Table 2: Linkage disequilibrium data for relevant *PTGS1* SNPs

| **Overall study group**  **(n= 113) D’, r** | **rs1236913** | **rs3842787** | **rs5787** | **rs3842792** |
| --- | --- | --- | --- | --- |
| **rs10306225** | 0.1923, 0.0935 | 0.9560, -0.033 | 0.9917, 0.6981 | 0.3171, -0.028 |
| **rs1236913** |  | 0.9987, 0.9298 | 0.8852, -0.0231 | 0.9870, 0.2384 |
| **rs3842787** |  |  | 0.8687, -0.0211 | 0.9871, 0.2561 |
| **rs5787** |  |  |  | 0.0033, 0.0023 |
| **Patients with late onset DILI**  **(n= 5) D’, r** | **rs1236913** | **rs3842787** | **rs5787** | **rs3842792** |
| **rs10306225** | 0.1665, -0.089 | 0.9994, -0.408 | 0.9996, 0.6121 | 0.9982, -0.2717 |
| **rs1236913** |  | 0.9996, 0.7635 | 0.9992, -0.3271 | 0.999, 0.5086 |
| **rs3842787** |  |  | 0.9988, -0.2497 | 0.9991, 0.6661 |
| **rs5787** |  |  |  | \| 0.9963, -0.1661 \|  \| \| --- \| --- \| |
| **Control subjects**  **(n= 100) D’, r** | **rs1236913** | **rs3842787** | **rs5787** | **rs3842792** |
| **rs10306225** | *.* | . | . | . |
| **rs1236913** |  | 0.9987; 0.9570 | . | . |
| **rs3842787** |  |  | . | . |
| **rs5787** |  |  |  | . |
